# Beyond Infrastructure: Shared Resources as Drivers of Impact and Collaboration in the NCI Cancer Center Community

**DOI:** 10.7171/001c.166268

**Published:** 2026-09-01

**Authors:** Brian C. Springer, Nicholas P. Ambulos, David M. Gosky

**Affiliations:** 1 Adminstration Moffitt Cancer Center; 2 Department of Microbiology and Immunology and Department of Medicine University of Maryland Marlene and Stewart Greenebaum Comprehensive Cancer Center; 3 Administration Fox Chase Cancer Center

**Keywords:** Shared Research Resources (SRR), Core facilities, NCI designated Cancer Center, research infrastructure, translational research, multi-institutional collaboration, financial stability

## Abstract

Shared Resources (SRs) are essential to advancing team science in NCI-Designated Cancer Centers by providing access to specialized expertise, advanced technologies, and state-of-the-art instrumentation. Effective SRs rely on strategic planning, continuous evaluation, infrastructure investment, and user feedback to remain responsive to evolving scientific needs. Beyond technical support, SRs strengthen institutional research capacity by fostering collaboration, leveraging resources, supporting external partnerships, and serving as hubs for mentorship and training. Regional and national efforts have also expanded multi-institutional SR networks, promoting greater efficiency, innovation, and collaboration. Integrating SRs into the broader research ecosystem creates a strong foundation for transdisciplinary discovery, institutional resilience, and sustained innovation amid changing funding environments.

## Introduction

Developing and operating efficient Shared Resources (SRs) is essential for effectively catalyzing biomedical team science.^1,2^ SRs enable scientists to conduct high-impact research within National Cancer Institute (NCI)-designated cancer centers using the most up-to-date techniques, equipment, and capabilities. Experienced SR leaders leverage strategic planning and evaluation, state-of-the-art facilities and equipment, and user feedback to remain agile in the face of emerging scientific challenges and opportunities. However, the impact of SRs extends far beyond technical support and scientific collaboration — SRs also galvanize institutional capabilities by leveraging resources across an institution and fostering external relations (e.g., industry partnerships). As intellectual hubs, SR staff members serve as mentors who bridge the gap between novel methodologies and their practical application in complex research. Additionally, these staff members develop talent pipelines for faculty, staff, and trainees. Regional and national efforts to enhance efficiency, collaboration, and innovation have fostered multi-institutional SR networks. Ultimately, embedding SRs into the broader ecosystem will create a foundational catalyst for innovation and transdisciplinary research within and among NCI-designated cancer centers while also adapting to extramural financial trends.^3^

## Role of Shared Resources in Innovation

SRs constitute more than just equipment, advice on experimental designs, and biostatistical/bioinformatic analyses; they are part of the bedrock of transformative team science. As a collective brain trust spanning the National Cancer Program, SRs provide the deep expertise and consultation necessary to guide investigators through complex experimental design, data interpretation, state-of-the-art technological applications, and collaborative internal and external workflows. Successful SR directors stay ahead of the curve by adopting technological breakthroughs that provide investigators with a distinct competitive edge. By fostering a user-centric environment wherein feedback is paramount, SRs can leverage investigator needs to further accelerate discovery. Ultimately, these facilities empower team science not only for individuals but also for the entire institution through inter-resource collaborations that leverage individual strengths and foster entirely new capabilities. The growth of SR networks in the national ecosystem has led to an increased ability to conduct impactful, multi-institutional research.

For example, spatial omics bridges the gap between molecular profiling and tissue architecture.^4,5^ However, implementing spatial omics requires more than just genomic or proteomic technology; it requires a strategic partnership with a pathology SR. In this model, pathology experts provide the critical tissue annotation and quality control necessary to transform raw spatial data into meaningful molecular and structural insights. Then, these capabilities can be offered both within an institution and can be made available to other institutions, thus enabling centers to focus on particular strategic opportunities without having to reproduce high-end equipment that may be available at partner centers.

## Role of Shared Resources in Therapeutic Development

Many types of SRs (e.g., biospecimen, biostatistics and informatics, biomarker, pharmacokinetics/pharmacodynamics, imaging, and population) provide an infrastructure for accelerating the development and translation of benchside discoveries into high-impact clinical trials. SRs help generate high-quality data that are required to initiate novel trials; furthermore, SRs provide specialized analytical platforms that are necessary for validating pharmacodynamic endpoints within investigator-initiated trials (IITs).^3^ A representative example of this synergy begins with the genomics SR identifying a novel biomarker associated with a specific malignancy. The biospecimen SR then facilitates a retrospective validation study by providing high-quality, longitudinal samples from its repository. These validated findings directly inform the design of a novel IIT, and the genomics SR provides the prospective molecular profiling necessary to confirm clinical utility in a new patient cohort. By bridging the gap between discovery and therapeutics, SRs facilitate an iterative discovery framework wherein clinical observations directly inform the next generation of mechanistic questions. This process is reflected in the acknowledgments and citations of numerous high-impact publications.

## Role of Shared Resources in Education

Each center’s Cancer Education & Training Coordination (CRTEC) unit provides a structured framework for mentorship and professional growth, empowering trainees — from high school students to early-career faculty investigators — with the leadership and career development tools necessary to sustain a lifelong trajectory in cancer research.^1,2^ The integration of SRs and CRTEC units reimagines these facilities as “learning laboratories.” In this ecosystem, the bidirectional flow of expertise between SR directors, staff, and trainees accelerates both individual career trajectories and institutional research throughput. Training is conducted for scientific methodology, research design, data analysis, technical use, and project management. While quantitative metrics confirm that trainees (e.g., undergraduate students, predoctoral fellows, postdoctoral fellows) are often key SR users, the value extends beyond mere usage. For early-career researchers, SRs provide a critical academic environment in which they transition from technical novices to independent investigators who are capable of seamlessly integrating advanced technologies into their research design.

This synergy is formalized through leadership alignment between SRs, CRTEC units, and T32/F32 training grant directors. Together, these stakeholders curate specialized curricula — including workshops, credit-bearing courses, and technology seminars — that transcend standard bench training. Furthermore, this environment fosters “shortcut mentorship,” such that experienced trainees troubleshoot technical hurdles and mentor peers, thus creating a self-sustaining cycle of peer-led innovation.

## Role of Shared Resources in Community Outreach & Engagement

The synergy between SR and community outreach & engagement (COE) teams transforms technical infrastructure into a tool for community impact.^6,7^ This synergy can enable individual scientists, teams, and programs to actively identify and address unique population health challenges in a center’s catchment area.^8^ Thus, SRs can serve as a vital bridge to the local community by providing services and participating in community-facing events.

For example, biospecimen SRs serve as stewards of ethically consented, patient-donated samples, thus maintaining the integrity of the trust placed in a center.^9,10^ Because these collections predominantly represent the local catchment area, they enable precision research that directly addresses the unique biological and health disparities of a specific subpopulation. Recruitment SRs serve as a catalyst between biorepository SRs and Clinical Trials Offices (CTOs). By streamlining the identification of eligible participants, these SRs facilitate the transition from specimen donation to clinical intervention. This synergy ensures that local patients are prioritized for both therapeutic and non-therapeutic trials, thereby maximizing the regional impact of a cancer center’s research.

Strategic synergies between SRs and COE teams are frequently realized through targeted infrastructure. For instance, population SRs provide the framework for rigorous behavioral and epidemiological research, while biostatistics and informatics SRs offer the analytical precision necessary to interpret complex community health data. Furthermore, these resources facilitate the development of digital health tools and mobile applications, thus bridging the gap for rural or homebound populations to ensure equitable access to cancer care.

## Linking Education and Community

As previously noted, SRs are vital educational cornerstones for cancer centers. By extending their reach beyond the laboratory, SRs serve as ambassadors of innovation, engaging the local population through targeted science, technology, engineering and mathematics (STEM) initiatives. In collaboration with CRTEC units, SRs can host immersive STEM demonstrations and hands-on experiences that provide tangible value to community members. These efforts transform SRs into “learning laboratories” that simplify complex research and foster a culture of “community science.” Furthermore, these activities impact the community at multiple levels by developing interest and excitement in cancer science, building talent pipelines, enhancing community relationships, fostering economic development, and ultimately improving cancer outcomes.

## Administration and Shared Resources in Planning and Evaluation

The effectiveness of SRs is fundamentally tied to a cancer center’s administrative infrastructure and policies. Maintaining a high-performing portfolio of SRs requires critical scientific planning, monitoring, and financial oversight. This strategic and “user-centric” model ensures that technological investments are directly responsive to investigator and center needs. By bridging SRM with a cancer center’s broader administration — a sophisticated network encompassing human resources, fiscal and grants management, clinical trials, education, publications, and space oversight — each center can ensure that technical advancements are matched by robust operational rigor and the meticulous tracking of key metrics, such as peer-reviewed funding and investigator-driven publications.

## Summary

SRs are not simply service providers; they are strategic, scientific pillars of NCI-designated cancer centers. By positioning SRs at the intersection of research, education, community impact, and clinical translation, cancer centers maximize their value to members, parent institutions, and the populations they serve. Additionally, through regional and national networks, SRs contribute to the National Cancer Program by fostering collaboration and efficiency, thus enabling cancer centers to focus resources on strategic efforts.

SRs not only facilitate data generation but also function as learning laboratories, thus bridging the gap between trainees and independent investigators as well as fostering a self-sustaining ecosystem of peer-to-peer learning. Specialized SRs — such as biorepositories, population sciences, and informatics SRs — position cancer centers as trusted stewards of community data, ensuring that local health needs are reflected in research outcomes. Furthermore, SRs catalyze a “bench-to-bedside and back” discovery framework, such that clinical trial observations directly inform the next generation of lab-based questions. The synergy between SRM and cancer center administrations ensures that scientific agility is always matched by operational rigor.

Rather than a static collection of equipment and staff, SRs ultimately serve as hubs for innovation by facilitating leading-edge capabilities, advanced equipment, expertise, and educational programs that yield exceptional science as well as collaboration with academic centers and industry partners. Throughout this journal, publications describe detailed work in these areas. The authors thus encourage continued investment in SRs, which provide efficient infrastructures and tremendous returns on investment for NCI-designated cancer centers.

### Conflict of Interest

The authors declare no competing financial interests.
